# Artifact Reduction Proton Density Magnetic Resonance Imaging Can Better Visualize Unicompartmental Knee Arthroplasty Components but Does Not Improve Measurement Accuracy at 3T: An In Vitro Phantom Study

**DOI:** 10.7759/cureus.46338

**Published:** 2023-10-01

**Authors:** Tsuneari Takahashi, Katsushi Takeshita

**Affiliations:** 1 Department of Orthopaedic Surgery, Ishibashi General Hospital, Shimotsuke, JPN; 2 Department of Orthopaedics, Jichi Medical University, Shimotsuke, JPN

**Keywords:** revision surgery, slice encoding for metal artifact correction, artifact reduction, mri, unicompartmental knee arthroplasty

## Abstract

Background

There are no studies of the efficacy of slice encoding for metal artifact correction (SEMAC) magnetic resonance imaging (MRI) at 3T for patients following unicompartmental knee arthroplasty (UKA), although the artifact is expected to increase compared with 1.5T.

Purpose

To clarify whether SEMAC MRI can better visualize UKA components and improve measurement accuracy at 3T MRI.

Materials and methods

The phantom consisted of femoral and tibial standard UKA components embedded in agarose gel. The MR images were scanned on a 3T MR system including proton density (PD) MR images. Six orthopedic surgeons blinded to the size and details of the components independently scored the diagnostic value for measurement and measured the lengths of the femoral posterior condyle, femoral peg, anterior-posterior (AP) tibial component, medial-lateral (ML) tibial component, and tibial keel, with and without SEMAC. Visualization scores were stratified as 0 = definitely nondiagnostic, 1 = probably nondiagnostic, 2 = possibly diagnostic, 3 = probably diagnostic, and 4 = definitely diagnostic. In addition, the differences between actual length and 95% confidence intervals of five measurement points were analyzed.

Results

The diagnostic values of the posterior condyle (2.0; 1.5 vs. 0; 0) and femoral peg (1.5; 1.0 vs. 0; 0) were significantly better in SEMAC-PD MRI than in non-SEMAC-PD MRI (P<0.05). On the other hand, there were no significant differences in the visualizations of AP, ML, and keel of the tibial components. Measurements of the femoral posterior condyle and tibial keel approached the actual length, but were not involved within the 95% confidence interval (actual length, 19.4 mm vs. 95% CI, 15.7-19.1 mm).

Conclusion

A significant reduction of metal artifacts was observed only around the femoral component in SEMAC-PD MRI. Despite artifact reduction, this sequence did not result in better visualization for measurement.

## Introduction

Osteoarthritis (OA) of the knee is one of the most common causes of painful loss of mobility in the middle-aged and elderly population [1]. OA is the main indication for knee joint replacement surgery. Unicompartmental knee arthroplasty (UKA) is a beneficial procedure for patients with degenerative OA limited to the medial or lateral compartment [2], providing reliable pain relief and improving function with significantly less morbidity and mortality compared with total knee arthroplasty [3]. However, well-functioning anterior cruciate ligament (ACL), medial collateral ligament (MCL), and preserved contralateral compartment are required for better clinical results and longer survival [4]. Therefore, there is a clinical need to accurately visualize the articular anatomy following UKA. Artifacts in magnetic resonance imaging (MRI) may be related to extrinsic factors, such as patient motion or metallic artifacts, and occur as a consequence of general image processing techniques [5]. Demand for improved techniques of MRI for postoperative evaluation is increasing [6,7]. Melcherczyk et al. reported that tailored MRI allows reproducible analysis of the component-bone interface following UKA. It is helpful for the assessment of suspected loosening following UKA [8]. Kleeblad et al. reported the efficacy of MRI with the addition of a multi-acquisition variable resonance image combination sequence, which is one of the metal artifact reduction sequences (MARS) for assessing symptomatic UKA and for quantifying appearances at the bone-component interface [9]. Agten et al. reported the efficacy of 1.5T MRI with slice encoding for metal artifact correction (SEMAC) in patients suffering pain following UKA and concluded that short-T1 inversion recovery (STIR)-SEMAC was useful for detecting bone marrow edema and influenced orthopedic surgeons' decisions about surgery [10]. MARS was first reported in 1994 [11], and SEMAC MRI was first reported by Lu et al. in 2009 [12]. SEMAC MRI uses two-dimensional slice selective excitations but then phase-encodes each slice in the through-plane dimension and combines them to form a composite image [10, 12]. SEMAC MRI corrects metal artifacts via robust encoding of each excited slice against metal-induced field inhomogeneities. This robust slice encoding is achieved by combining a VAT spin-echo sequence with additional z-phase encoding. Although the VAT compensation gradient works to suppress in-plane distortions, the z-phase encoding resolves the distorted excitation profiles that cause through-plane distortions. By positioning all spins in a region of interest to their actual spatial locations, the through-plane distortions can be corrected by summing up the resolved spins in each voxel. The SEMAC technique does not require additional hardware and can be easily deployed with existing whole-body MRI systems.

There are currently no studies of the efficacy of SEMAC MRI at 3T for patients following UKA, although the artifact is expected to increase at 3T compared with 1.5T due to enhanced field inhomogeneities and is known to differ depending on hardware composition [13,14]. Therefore, we conducted an in vitro experimental study to clarify whether SEMAC MRI can better visualize UKA components and improve the measurement accuracy of 3T MRI.

## Materials and methods

This in vitro experimental study was conducted in the Department of Orthopedic Surgery of a single institution. Approval of the institutional review board of the ethics committee of our institution was waived because the study was conducted without human specimens.

**Figure 1:**
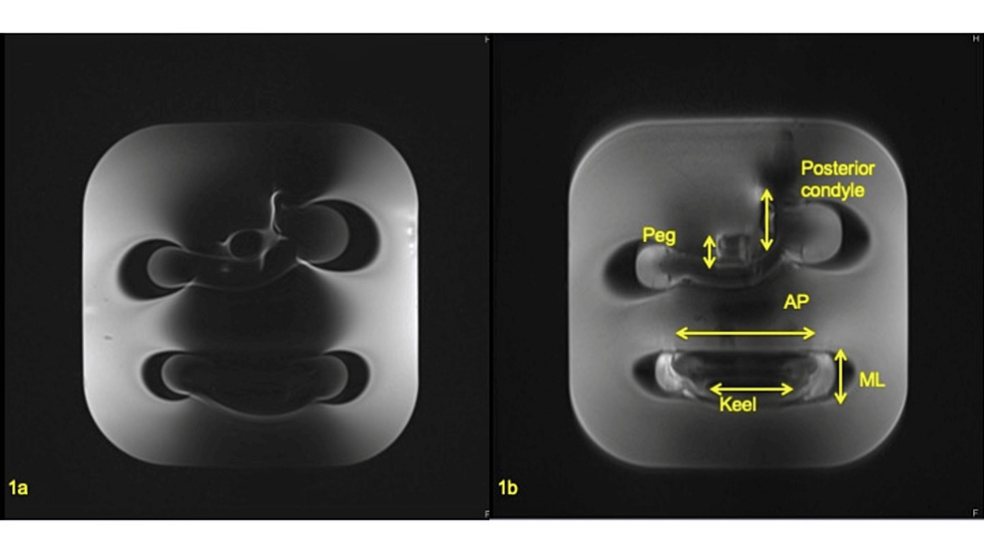
Images of the phantom scanned on 3T conventional proton density (PD) and (a) slice encoding for metal artifact correction (SEMAC) proton density magnetic resonance imaging (MRI). (b) Artifact reduction adjacent to the components was confirmed in Figure 1b. AP, anterior-posterior; ML, medial-lateral

In an in vitro experimental setup, the phantom consisted of femoral and tibial standard UKA components (Oxford partial knee arthroplasty phase 3, Zimmer Biomet, UK) embedded in agarose gel in a standard fashion [15]. The MR images were scanned on a 3T MR system (SEMENS, Echelon, Germany) including SEMAC-corrected and -uncorrected proton density (PD) MR images (Figure 1) [16].

PD sequence was used because this sequence could detect postoperative contralateral compartmental meniscal lesions [17] and predict implant loosening [18]. The Repetition time was 2467 ms. Echo time was 25 ms. The thickness of the slices was 3 mm, the gap was set at 0 and the acquisition time was 3 mins and 48 seconds [19]. Six board certified orthopedic surgeons who were blinded to the size and details of the components were included in this study as evaluators. The authors were not included in these six surgeons. They independently scored the diagnostic values for measurement and measured the lengths of the femoral posterior condyle, femoral peg, anterior-posterior (AP) tibial component, medial-lateral (ML) tibial component, and tibial keel, with and without SEMAC. The scores of component visualization were stratified as 0 = definitely nondiagnostic, 1 = probably nondiagnostic, 2 = possibly diagnostic, 3 = probably diagnostic, and 4 = definitely diagnostic [18]. In addition, the difference between the actual length measured by the manufacturer and 95% confidence intervals of five measurement points were analyzed.

Statistical analysis

The data are presented as means and standard deviations. Values of P < 0.05 were considered indicative of statistical significance. All statistical analyses were performed using EZR software (http://www.jichi.ac.jp/saitama-ct/SaitamaHP.files/statmed.html, Saitama Medical Center, Jichi Medical University, Saitama, Japan) [20]. Comparisons of nonparametric data were evaluated by the Mann-Whitney U test. The 95% confidence intervals (CIs) of each measured length were also calculated.

## Results

The diagnostic values of posterior condyle (2.0; 1.5 vs. 0; 0) and femoral peg (1.5; 1.0 vs. 0; 0) were significantly better in SEMAC-PD MRI than in non-SEMAC-PD MRI (P<0.05). On the other hand, there were no significant differences in the visualizations of AP, ML, and keel of the tibial component (Table 1).

**Table 1:** Visualization of embedded components Results of independent scoring of the diagnostic values for measurement and measured the lengths of the femoral posterior condyle, femoral peg, anterior-posterior (AP) tibial component, medial-lateral (ML) tibial component, and tibial keel, with and without SEMAC. The scores of component visualization were stratified as 0 = definitely nondiagnostic, 1 = probably nondiagnostic, 2 = possibly diagnostic, 3 = probably diagnostic, and 4 = definitely diagnostic. AP, anterior-posterior; ML, medial-lateral; CONV, conventional; SEMAC, slice encoding for metal artifact correction. Data are expressed as median (interquartile range).

		CONV	SEMAC	P value
Femur	Posterior condyle	0 (0)	2.0 (1.5)	<0.05
	Peg	0 (0)	1.0 (1.0)	<0.05
Tibia	AP	1.0 (0)	2.0 (1.5)	0.17

Measurements of the femoral posterior condyle and tibial keel approached the actual length, but were not involved within the 95% CI (actual length, 19.4 mm vs. 95% CI, 15.7-19.1 mm) (Table 2).

**Table 2:** Differences between actual length and results of measurement in SEMAC-PD Results of the differences between actual length measured by the manufacturer and 95% confidence intervals. AP, anterior-posterior; CI, confidence interval; SEMAC-PD, slice encoding for metal artifact correction

		Actual (mm)	Mean (mm)	95% CI (mm)
Femur	Posterior condyle	19.4	17.4	15.7-19.1
	Peg	18.5	12.4	11.3-13.4
Tibia	AP	45.2	60.7	56.8-64.6

## Discussion

Our data showed that a significant reduction of the metal artifacts was only observed around the femoral component in SEMAC-PD MRI at 3T. Despite artifact reduction, this sequence did not result in better visualization for measurement.

Sutter et al. compared SEMAC versus optimized standard MRI in patients following total knee arthroplasty and described how the use of SEMAC sequences resulted in a statistically significant reduction in artifacts at 1.5T [21]. The detection of clinically relevant findings such as periprosthetic osteolysis was markedly improved [21], which is in accordance with the results of our study. SEMAC MRI reduces metal-induced artifacts. However, this study did not describe how SEMAC improved the accuracy of measurement of the length around the total knee arthroplasty components despite the non-inferiority that Liebl et al. described that metal artifact reduction is feasible at 3T and similarly effective compared to lower field strengths, which is particularly important given today’s increasingly widespread use of 3T high-field MRI [19]. One of the plausible reasons was that PD-SEMAC suffers from blurring of images, potentially masking relevant meniscal lesions [10]. PD-SEMAC does not improve cartilage lesion detection in the non-operated compartments [10]. However, visualization of the UKA component-bone interface was not described in that study. According to the study of Baker et al., 23% of UKA revision surgeries are due to unexplained pain, as shown by data from the National Joint Registry of England and Wales in the period from 2003 to 2010 [22]. Therefore, the reason that we undertook this in vitro experiment in PD sequence is as follows. Intact ACL, MCL, and contralateral component are prerequisites for the longevity of well-functioning UKA. Therefore, during the follow-up period after surgery, adequate MARS sequence to visualize these structures is required. The signal generated by PD sequences depends principally on the number of protons within the tissue. The signal is sensitive to tears and degenerative changes in the ACL and menisci that may result in poorer postoperative patient-reported outcomes [23]. In addition, even though the measurement accuracy is low, SEMAC MRI could reduce the artifact around the UKA components, thus enabling analysis of the component-bone interface following UKA. It is helpful in the assessment of suspected loosening following UKA, and therefore further study will be required to obtain better visualization using this MARS technique.

This study had several limitations. First, phantom was used, and this study was not conducted on human patients. Because it is an in vitro phantom study, results may not directly reflect performance in human patients. On the other hand, phantom-based studies can be evaluated without the blurring caused by patient motion during MRI imaging. Future in vivo human studies are required. Second, only one design and size UKA was used in this study due to the limited availability of the implants. Results may vary with different implants. Third, the sample size is small. With six evaluators, statistical power may be limited. Fourth, artifact size was measured in-plane only. Above these limitations, however, this study clarified that SEMAC MRI could better visualize UKA components but did not improve measurement accuracy at 3T MRI.

## Conclusions

Even though the measurement accuracy is low, SEMAC MRI could reduce the artifact around the UKA components, thus enabling analysis of the component-bone interface following UKA. It is helpful in the assessment of suspected loosening following UKA, and therefore further study will be required to obtain better visualization using this MARS technique. The effect of artifact reduction in SEMAC-PD MRI was only evident in the femoral component at 3T. SEMAC-PD MRI with the motion blur that may occur in living patients may not be feasible for measuring cysts and avulsions adjacent to the UKA at 3T.
